# Monitoring of *JAK2*V617F allele burden in patients with essential thrombocythemia

**DOI:** 10.1007/s44313-026-00137-2

**Published:** 2026-04-22

**Authors:** Jeong Suk Koh, Wonhyoung Seo, Sora Kang, Myung-Won Lee, Hyewon Ryu, Seung-Woo Baek, Ik-Chan Song, Hyo-Jin Lee, Deog-Yeon Jo

**Affiliations:** Division of Hematology/Oncology, Department of Internal Medicine, Chungnam National University College of Medicine, 282 Munhwa-Ro, Jung-Gu, Daejeon, 35015 Korea

**Keywords:** Essential thrombocythemia, *JAK2*V617F allele burden, Transformation-free survival

## Abstract

**Background/Aim:**

This study retrospectively examined the clinical implications of monitoring *JAK2*V617F, with a particular focus on disease transformation, in a Korean population of patients with essential thrombocythemia (ET).

**Methods:**

Medical records of patients diagnosed with ET between January 1996 and December 2021 at Chungnam National University Hospital, Daejeon, Korea, were reviewed. Both episodic changes (increase or decrease) and longitudinal patterns of change (stable, gradual increase, or gradual decrease) in *JAK2*V617F variant allele frequency (VAF), measured at 1-year intervals, were analyzed.

**Results:**

Among the 87 patients who had *JAK2*V617F VAF measured at least three times, 23 (26.4%), 21 (24.1%), and 16 (18.4%) experienced increases of ≥ 25%, ≥ 50%, and ≥ 100%, respectively, while 27 (42.5%), 26 (29.9%), and 2 (2.3%) experienced decreases of ≥ 25%, ≥ 50%, and 100%, respectively. Patients with VAF increases had significantly poorer transformation-free survival than those without increases (15-year survival for ≥ 50% increase: 79.2% *vs.* 97.5%; *p* = 0.007). Regarding the longitudinal patterns, 62 (71.3%), 13 (14.9%), and 12 (13.8%) patients were classified as having stable, gradual increase, and gradual decrease patterns, respectively. Transformation-free survival was worse in patients with a gradual increase and better in those with a gradual decrease than in those with a stable pattern (20-year survival: 100% *vs.* 88.8% *vs.* 31.3%; *p* = 0.026).

**Conclusions:**

An increasing *JAK2*V617F allele burden over time is associated with disease transformation in ET, supporting the need for the ongoing monitoring of *JAK2*V617F VAF in these patients.

## Introduction

Philadelphia chromosome–negative myeloproliferative neoplasms (Ph^−^ MPNs) are clonal hematological disorders that include essential thrombocythemia (ET), polycythemia vera (PV), and primary myelofibrosis. These disorders are characterized by increased blood cell counts, frequent thrombotic vascular events [1], and myelofibrotic or leukemic transformation [2]. Thrombotic vascular events in patients with Ph^−^ MPNs often lead to substantial morbidity and mortality. Arterial events occur more frequently than venous ones, and acute coronary syndrome and cerebral infarction are the most common manifestations [1, 3–6].

*JAK2*V617F is the most commonly detected driver mutation in patients with ET [1] and is a well-known risk factor for thrombosis [7, 8]. Accordingly, *JAK2*V617F has been incorporated into thrombotic risk stratification for ET [9]. A higher *JAK2*V617F allele burden confers a higher risk of thrombosis in patients with Ph^−^ MPNs [10, 11] and is associated with progression to myelofibrosis [12, 13]. Thus, monitoring the allele burden in these patients has been suggested [13]. Additionally, reductions in the mutated *JAK2* allele burden have been associated with favorable clinical outcomes in patients treated with Ph^−^ MPNs [14]. Collectively, these observations suggest that the *JAK2*V617F allele burden is emerging as an important biomarker for predicting the clinical course of patients with Ph^−^ MPNs.

The clinical features of Korean patients with Ph^−^ MPNs differ from those of Western populations. For example, the pattern of thrombotic events varies; arterial thrombosis is far more common than venous thrombosis, ischemic stroke occurs more frequently, and deep vein thrombosis is much less common [1]. In this context, the clinical implications of monitoring the mutated *JAK2*V617F allele burden in Korean patients with Ph^−^ MPNs remain limited and require further clarification. In this study, we retrospectively evaluated *JAK2*V617F allele burden at diagnosis and the changes therein during the clinical course, focusing on its relationship with thrombosis and disease progression in a Korean cohort of patients with ET.

## Methods

### Patients

We reviewed the medical records of patients diagnosed with ET between January 1996 and December 2021 at the Chungnam National University Hospital, Daejeon, Korea. For patients diagnosed before 2017, the diagnosis was reassessed according to the 2016 World Health Organization (WHO) criteria [15]. Demographic and laboratory data, including complete blood counts, blood chemistry, and driver gene mutations, were collected from medical records. Patients with the *JAK2*V617F mutation were included in this study. Before 2016, *JAK2*V617F analysis was performed to confirm the diagnosis. After 2016, *JAK2*V617F variant allele frequency (VAF) was measured annually, following recommendations to monitor allele burden in patients with Ph^−^ MPNs [13]. Prognostic stratification was performed using the International Prognostic Score for Essential Thrombocythemia (IPSET) [16]. Hydroxyurea or anagrelide was used for cytoreduction, based on standard recommendations, drug availability, and patient compliance. Except for patients classified as low or very low risk, low-dose aspirin (100 mg daily) was prescribed to prevent thrombosis (Table 1).

**Table 1:** Patient characteristics (*n* = 106)

Characteristics at diagnosis	
Age, years	66 (18 − 84)
Male sex	48 (45.3)
Time of diagnosis	
1996 − 2015	40 (37.7)
2016 − 2022	66 (62.3)
Palpable splenomegaly	0 (0.0)
Volumetric splenomegaly (*n* = 75)	36 (48.0)
Laboratory findings	
WBCs, × 10^9^/L	11.3 ± 4.2
Neutrophils/lymphocytes	4.1 ± 2.2
Monocytes, × 10^9^/L	0.7 ± 0.5
Hemoglobin, g/dL	13.9 ± 1.8
Platelets, × 10^9^/L	840.6 ± 319.1
LDH, × ULN	1.2 ± 0.4
*JAK2*V617F VAF, % (*n* = 91)	24.7 ± 13.8
IPSET	
Low	25 (23.6)
Intermediate	30 (28.3)
High	51 (48.1)
Comorbidity	
Hypertension	46 (43.4)
Diabetes mellitus	16 (15.1)
Chronic kidney disease	10 (9.4)
Dyslipidemia	20 (18.9)
Smoking	18 (20.0)
Treatment at diagnosis	
Cytoreductive treatment	80 (75.5)
Aspirin	100 (94.3)
Initial thrombotic events	
Time of occurrence	
Before or at diagnosis	13 (12.2)
After diagnosis	14 (13.2)
Overall	27 (25.4)
Vessels involved	
Arterial	26 (24.5)
Venous	1 (1.0)
Follow-up, years	6.2 (0.6 − 29.5)

Data are presented as median (range), n (%), or mean ± standard deviation

*WBC* white blood cell, *LDH* lactate dehydrogenase, *ULN* upper limit of normal, *VAF* variant allele frequency, *IPSET* International Prognostic Score for Essential Thrombocythemia

### *JAK2* mutation analyses

*JAK2*V617F was detected using polymerase chain reaction and Sanger sequencing before 2010, after which allele-specific real-time quantitative polymerase chain reaction was used. Among patients who underwent at least three VAF measurements during the study period, individual episodes of VAF change and the overall temporal pattern were analyzed. For episodic changes, VAF variations from baseline were categorized into six groups: ≥ 25% increase, ≥ 50% increase, ≥ 100% increase, ≥ 25% decrease, ≥ 50% decrease, and 100% decrease, regardless of treatment status. Episodic changes in *JAK2*V617F levels may fluctuate in the same patient, which can lead to an overlap of group categorization. Conversely, some patients may not belong to any group if no change in the VAF is observed. The overall pattern of the VAF change was classified into three categories: gradual increase, gradual decrease, or stable. A “gradual increase” was defined as a ≥ 25% rise occurring at least twice during follow-up. A “gradual decrease” was defined similarly. Cases not meeting either definition were classified as “stable.”

### Definition of splenomegaly

Splenomegaly was defined according to the previously described criteria [17]. “Palpable splenomegaly” indicated a spleen that was palpable below the left costal margin. “Volumetric splenomegaly” was defined as a spleen volume exceeding the mean plus three standard deviations of the reference volume for the patient’s age and body surface area.

### Definitions of thrombotic vascular events and comorbidity

Thrombotic vascular events included cerebrovascular events (ischemic stroke, transient ischemic attack, and venous sinus thrombosis), coronary events (acute coronary syndrome), and splanchnic and peripheral thromboembolisms. Events that occurred before diagnosis, at the time of diagnosis, and during follow-up were included. The comorbidities included hypertension, diabetes mellitus, chronic kidney disease, dyslipidemia, and smoking.

### Definitions of myelofibrotic and leukemic transformations

Myelofibrotic transformation was diagnosed according to the 2016 WHO criteria [15]. Leukemic transformation (to acute myeloid leukemia) was defined as the presence of ≥ 20% blasts in the peripheral blood or bone marrow.

### Statistical analysis

Descriptive data are presented as mean ± standard deviation, median (range), or percentage, and were analyzed using Student’s t-test or the chi-square test, as appropriate. Correlations between *JAK2*V617F VAF and other variables were evaluated using Pearson’s correlation analysis. Survival was estimated using the Kaplan–Meier method and compared using the log-rank test. Overall survival, thrombosis-free survival, and transformation-free survival were defined as the times from ET diagnosis to death from any cause, thrombosis, or progression to myelofibrosis or leukemia, respectively. The risk factors for survival were assessed using Cox regression analysis. Statistical analyses were performed using SPSS version 24.0 (IBM Corp., Armonk, NY, USA), and *p* < 0.05 was considered statistically significant.

### Ethics

The study was conducted in accordance with the Declaration of Helsinki and approved by the Institutional Review Board (IRB) of Chungnam National University Hospital (IRB No. 2025–12–018). The requirement for informed consent was waived by the IRB because of the retrospective nature of the study. This research was supported by a grant from Chungnam National University. The funder had no role in the study design, data collection and analysis, decision to publish, or manuscript preparation.

## Results

### Patient characteristics

During the study period, 176 patients were newly diagnosed with ET. Of these, 106 (60.2%) patients with the *JAK2*V617F mutation, with a median age of 66 years (range, 18–84 years), were included. They were followed for a median of 6.2 years (range, 0.6–29.5 years). Based on the IPSET, high-risk patients were the most common (48.1%), followed by intermediate-risk (28.3%) and low-risk (23.6%) patients. Cytoreductive therapy was prescribed to 80 (75.5%) patients at diagnosis. Most patients were administered low-dose aspirin. Thrombotic vascular events occurred in 27 patients (25.5%), half of whom occurred shortly before or at the time of diagnosis. Arterial events (24.5%) were far more common than venous events (1.0%) (Table 1).

### *JAK2*V617F allele burden at the time of ET diagnosis

**Fig. 1:**
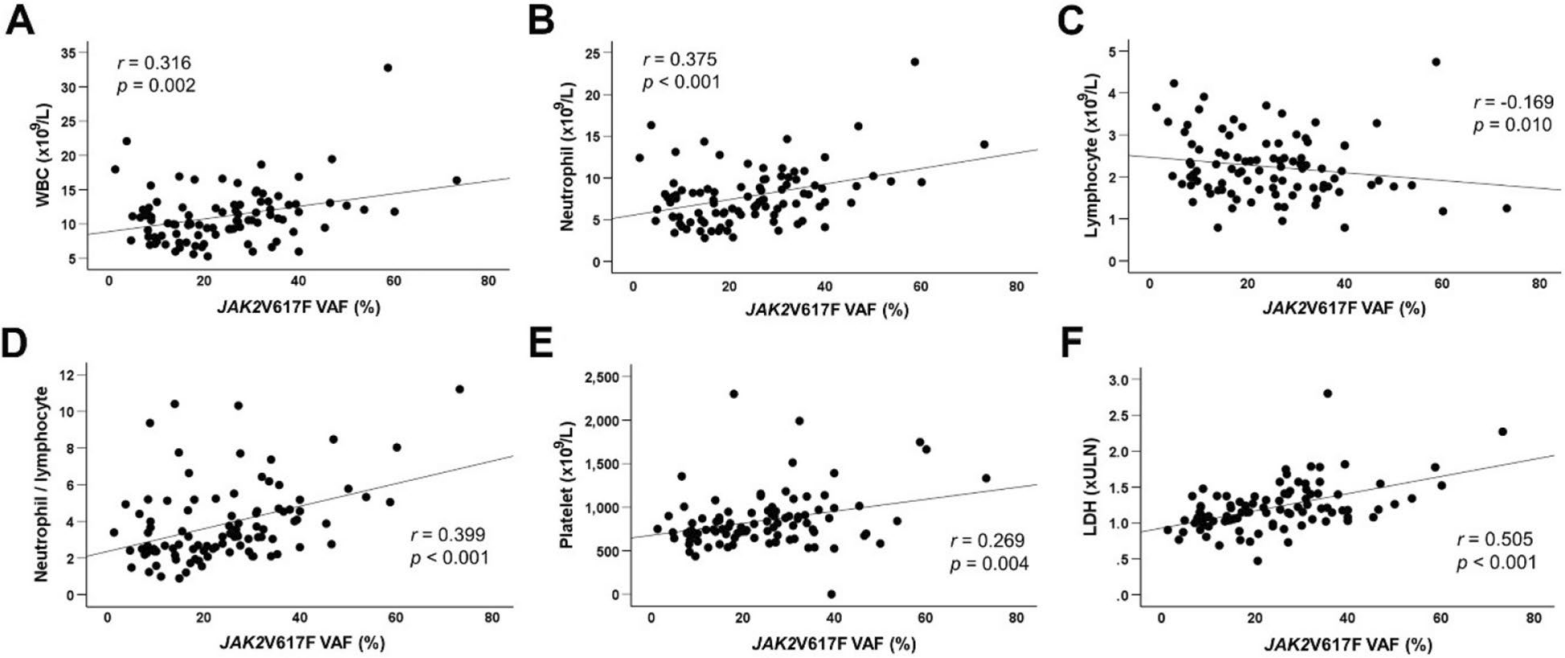
Correlations between *JAK2*V617F VAF and various parameters (*n* = 91). *VAF* variant allele frequency, *WBC* white blood cell, *LDH* lactate dehydrogenase

**Fig. 2:**
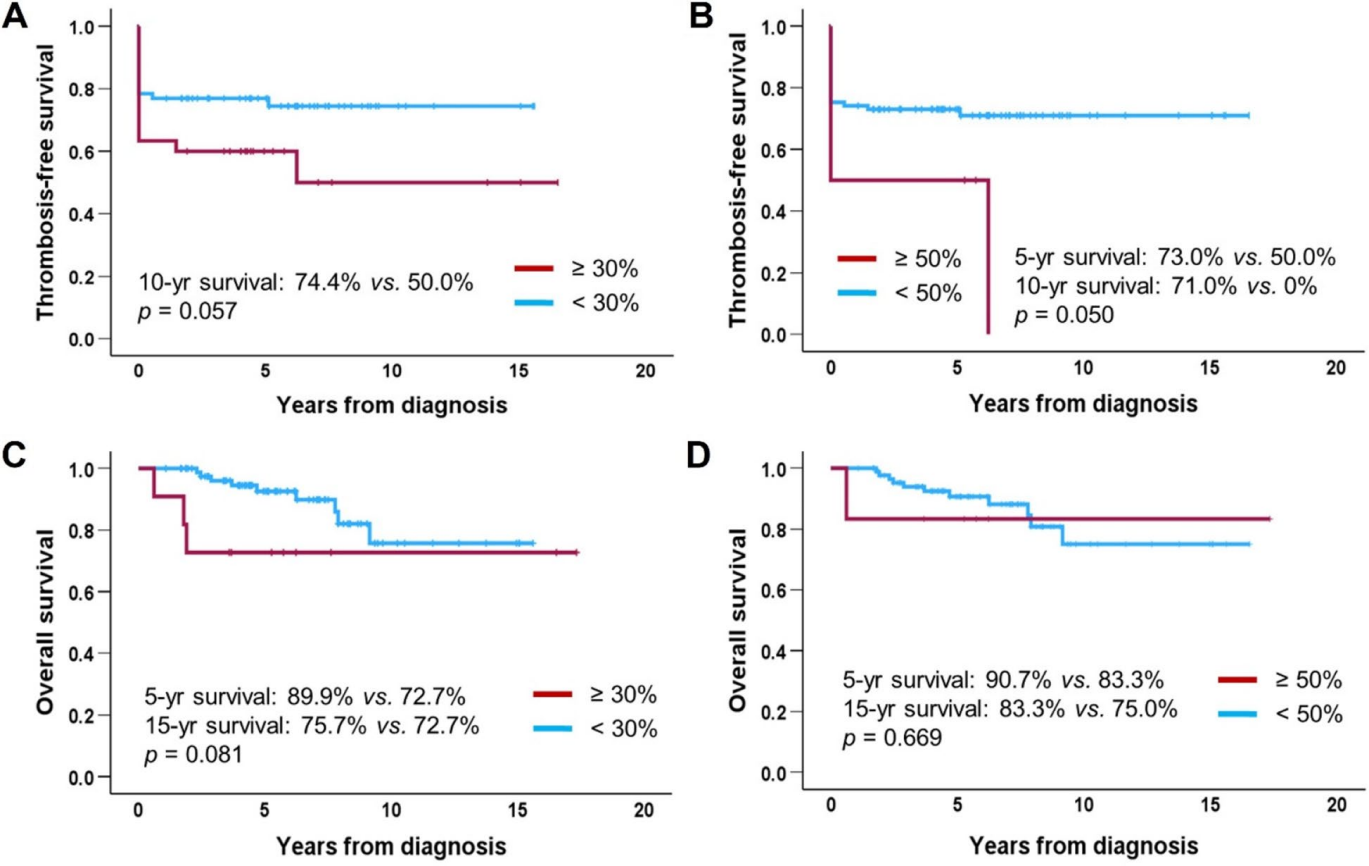
**A**, **B** Thrombosis-free survival and (**C**, **D**) overall survival according to *JAK2*V617F variant allele frequency at time of diagnosis in patients with essential thrombocythemia (*n* = 91)

*JAK2*V617F testing at diagnosis was performed in 91 (85.8%) of the 106 patients. The initial *JAK2*V617F VAF was 24.7% ± 13.8%. VAF was positively correlated with white blood cell count (*r* = 0.316; *p* = 0.002), neutrophil count (*r* = 0.375; *p* < 0.001), neutrophil-to-lymphocyte ratio (*r* = 0.339; *p* < 0.001), platelet count (*r* = 0.296; *p* = 0.004), and lactate dehydrogenase (LDH) normalized ratio (*r* = 0.505, *p* < 0.001). It was negatively correlated with lymphocyte count (*r* = − 0.169; *p* = 0.010) (Fig. 1). Patients with higher VAF tended to have poorer thrombosis-free survival. Using cutoffs of 30% and 50% VAF, the 10-year thrombosis-free survival rates were 74.5% *vs.* 50.0% (*p* = 0.057) and 71.0% *vs.* 0.0% (*p* = 0.050), respectively. Overall survival did not differ according to VAF levels (Fig. 2). Because only three transformation events (two myelofibrosis and one acute myeloid leukemia) were observed, transformation-free survival according to the VAF was not analyzed.

### Changes in *JAK2*V617F allele burden induced by hydroxyurea

**Fig. 3:**
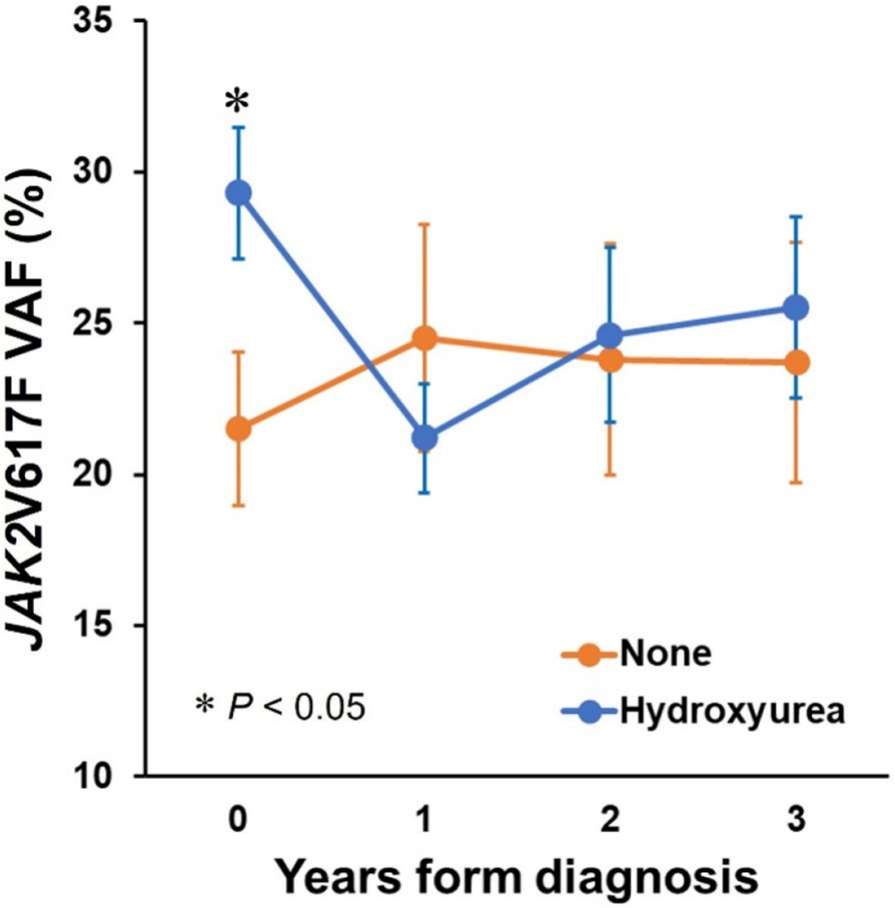
Changes in *JAK2*V617F VAF according to hydroxyurea treatment with time (*n* = 57). *VAF* variant allele frequency

Of the 106 patients, 57 (53.8%) underwent annual VAF measurements for 3 consecutive years after diagnosis. Among them, 39 received hydroxyurea, whereas 18 did not. At diagnosis, VAF was higher in treated patients than in untreated patients (29.3% ± 13.5% *vs.* 21.5% ± 10.7%; *p* = 0.036). In treated patients, VAF significantly decreased after 1 year (29.3% ± 13.5% to 21.2% ± 11.2%; *p* < 0.001) but then showed a modest rebound (24.6% ± 18.1% at year 2 and 25.5% ± 18.7% at year 3). In contrast, the VAF did not significantly change in untreated patients during the same period (Fig. 3).

### Episodes of changes in *JAK2*V617F VAF

**Fig. 4:**
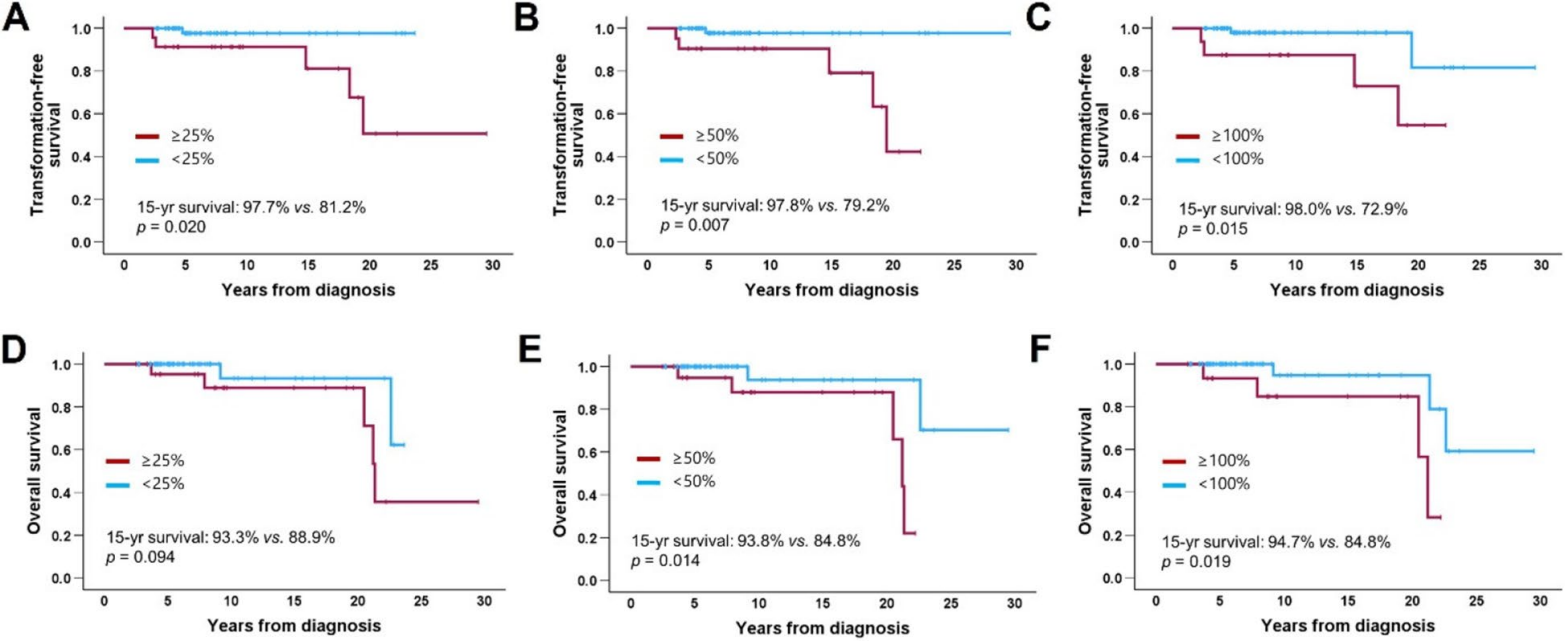
**A**–**C** Transformation-free survival and (**D**–**F**) overall survival according to the degree of increase in *JAK2*V617F variant allele frequency

The VAF was measured at least three times in 87 patients (82.1%). Of these, 23 (26.4%), 21 (24.1%), and 16 (18.4%) patients experienced at least one VAF increase of ≥ 25%, ≥ 50%, and ≥ 100%, respectively. Conversely, 27 (42.5%), 26 (29.9%), and 2 (2.3%) experienced at least one decrease of ≥ 25%, ≥ 50%, and 100%, respectively (Table 2). Patients who experienced any increase showed significantly poorer transformation-free survival than those who did not (15-year survival for ≥ 25% increase: 81.2% *vs.* 97.7%, *p* = 0.020; ≥ 50% increase: 79.2% *vs.* 97.5%, *p* = 0.007; ≥ 100% increase: 72.9% vs. 98.0%, *p* = 0.015) (Fig. 4), although thrombosis-free survival was unaffected (data not shown). Patients with increases of ≥ 50% and ≥ 100% also had worse overall survival (≥ 50%: 84.8% *vs.* 93.8%, *p* = 0.014; ≥ 100%: 84.8% *vs.* 94.7%, *p* = 0.019) (Fig. 4). A decrease in the VAF was not associated with survival outcomes.

**Table 2:** Changes in *JAK2*V617F variant allele frequency (*n* = 87)

	Patients
Episode of change	
Increase	
≥ 25%	23 (26.4)
≥ 50%	21 (24.1)
≥ 100%	16 (18.4)
Decrease	
≥ 25%	37 (42.5)
≥ 50%	26 (29.9)
100%	2 (2.3)
Pattern of change	
Stable	62 (71.3)
Gradual increase	13 (14.9)
Gradual decrease	12 (13.8)

Data are presented as n (%)

### Patterns of changes in *JAK2*V617F VAF

**Fig. 5:**
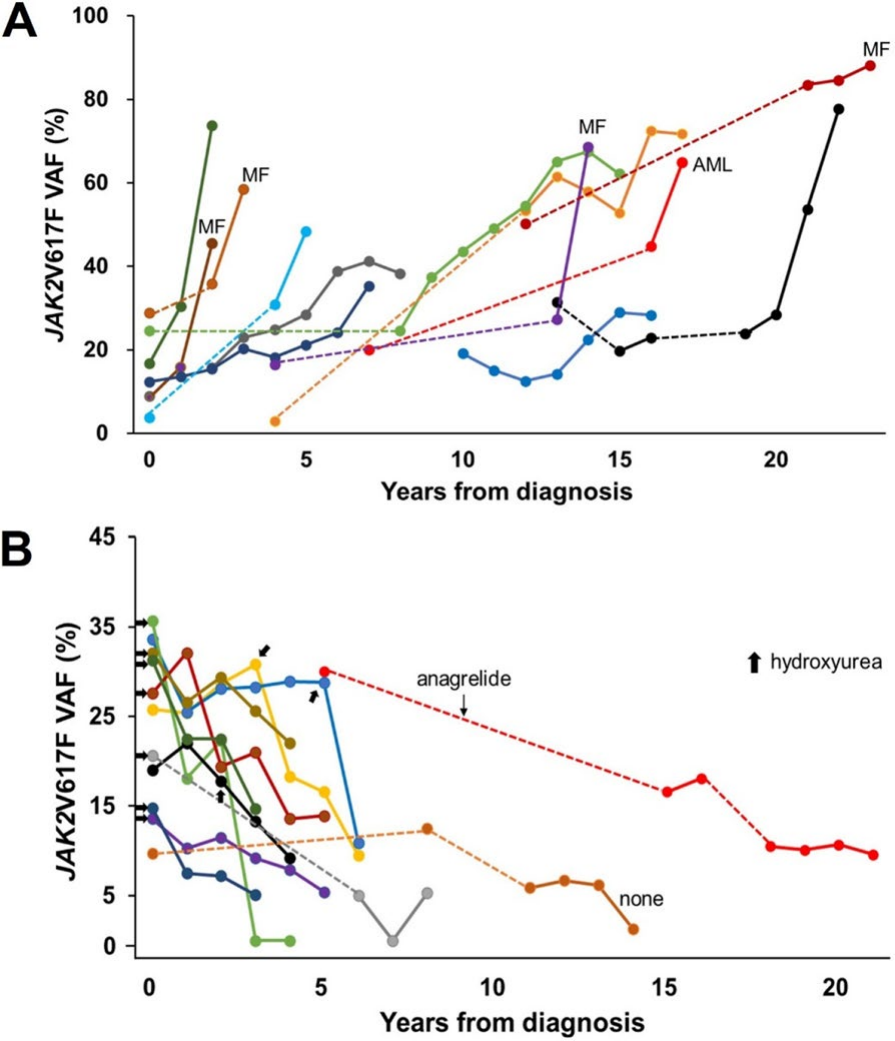
Changes in *JAK2*V617F VAF with time. **A** Patients with a gradual increase in VAF; (**B**) Patients with a gradual decrease in VAF. The dotted lines are imaginary ones that connect the measurements of two time points. *VAF* variant allele frequency

**Fig. 6:**
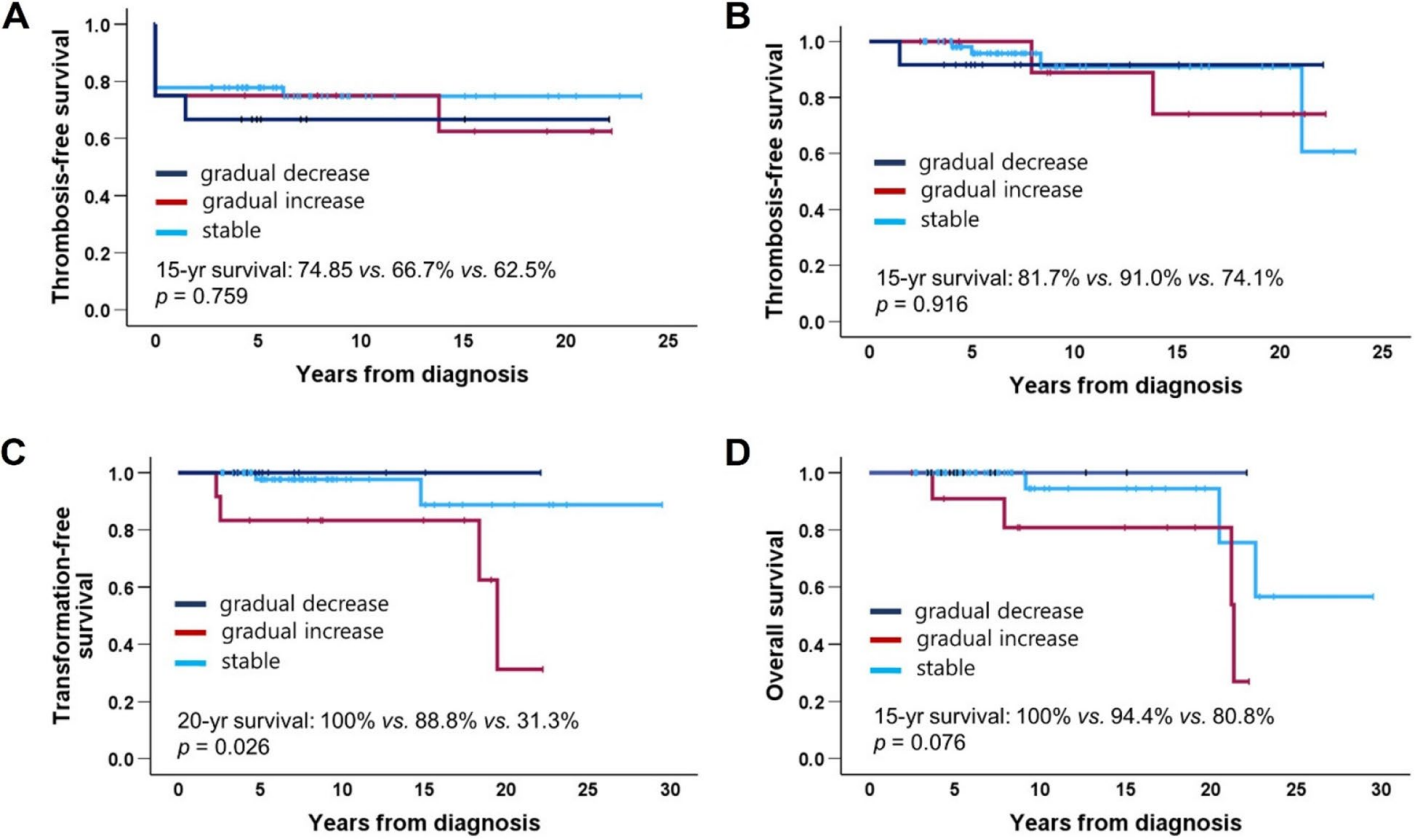
Survival according to pattern of changes in *JAK2*V617F variant allele frequency. **A** Overall thrombosis-free survival; **B** Post-diagnosis thrombosis-free survival; **C** Transformation-free survival; **D** Overall survival

Among 87 patients, 62 (71.3%) exhibited a stable pattern, 13 (14.9%) showed a gradual increase, and 12 (13.8%) showed a gradual decrease (Table 2 and Fig. 5). None of the patients with a gradual decrease received JAK inhibitors or interferons. In one, *JAK2*V617F was undetectable 3 years after diagnosis, and in another, it was undetectable 7 years after diagnosis (Table 3). Both patients had been treated with hydroxyurea since diagnosis. An 18-year-old male patient initially diagnosed with a platelet count of 806 × 10^9^/L showed a gradual decrease in VAF (from 10.0% to 1.0%) over 14 years without cytoreductive therapy, and his platelet count normalized by his last visit (Table 3). Thrombosis-free survival did not differ between the groups. However, transformation-free survival differed significantly; patients with a gradual increase had worse outcomes than those with stable patterns, whereas those with gradual decreases had better outcomes (20-year survival: 31.3% *vs.* 88.8% *vs.* 100%; *p* = 0.026). A similar trend was observed for overall survival, although the difference was not significant (Fig. 6). VAF at transformation (n = 6) was significantly higher than VAF at diagnosis (n = 91) (59.5% ± 19.8% *vs.* 24.7% ± 13.8%; *p* < 0.001).

**Table 3:** Representative clinical features of patients who had a gradual decrease in *JAK2*V617F allele burden

	Patient 1		Patient 2		Patient 3	
	Diagnosis	Follow-up	Diagnosis	Follow-up	Diagnosis	Follow-up
Age, years	18	32	73	80	76	79
IPSET	Low	−	High	−	High	−
Cytoreduction	None	None	Hydroxyurea	Hydroxyurea	Hydroxyurea	Hydroxyurea
*JAK2*V617F VAF, %	10.0	1.0	21.0	0.0	35.7	0.0
WBCs, × 10^9^/L	7.1	8.3	9.4	4.5	10.8	6.1
Hemoglobin, g/dL	16.0	16.1	15.7	11.2	13.6	12.0
Platelets, × 10^9/^L	806.0	418.0	722.0	178.0	537.0	240.0
LDH, × ULN	1.1	1.4	0.5	0.5	2.8	0.9
Thrombosis	None	None	Cerebral infarction	None	None	None
Transformation	−	None	−	None	−	None

*IPSET* International Prognostic Score for Essential Thrombocythemia, *VAF* variant allele frequency, *WBC* white blood cell, *LDH* lactate dehydrogenase, *ULN* upper limit of normal

### Risk factors for survival

**Fig. 7:**
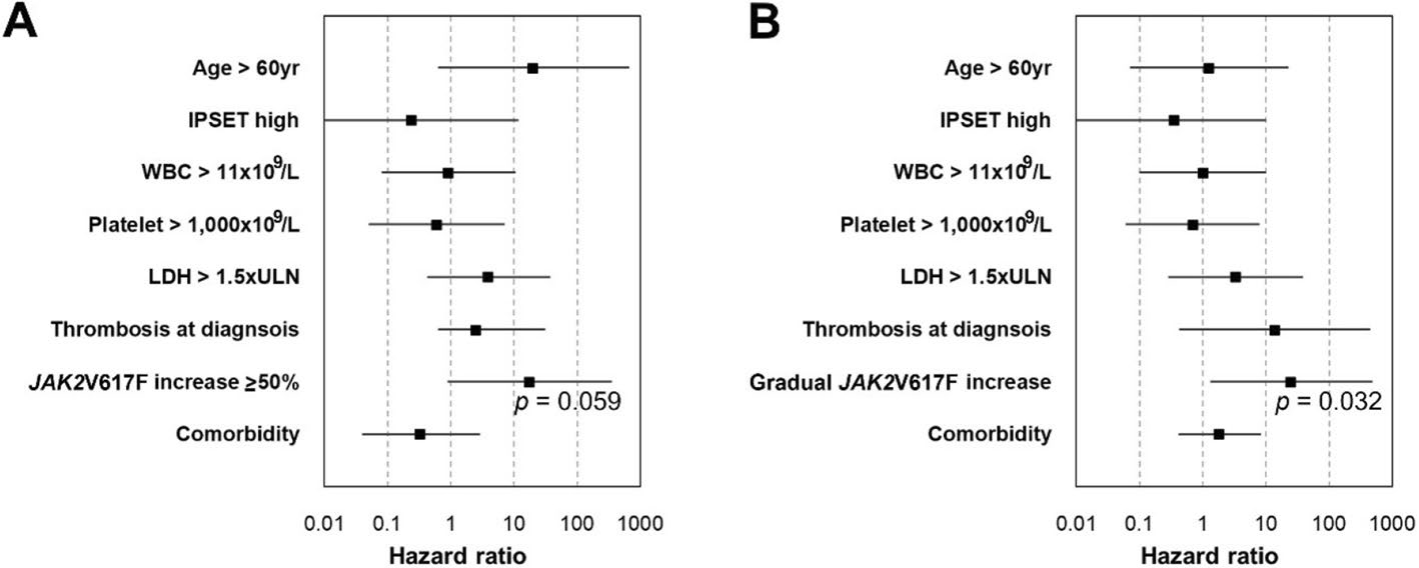
Multivariate analysis of risk factors for transformation-free survival. **A** Analysis including an increase in *JAK2*V617F variant allele frequency (VAF) by ≥ 50%; **B** Analysis including a gradual increase in *JAK2*V617F VAF. *IPSET* International Prognostic Score for Essential Thrombocythemia, *VAF* variant allele frequency, *WBC* white blood cell, *LDH* lactate dehydrogenase, *ULN* upper limit of normal

Comorbidity was the only risk factor for post-diagnosis thrombosis-free survival in the univariate analysis (hazard ratio [HR], 10.39; 95% confidence interval [CI], 1.12–88.03; *p* = 0.032). An increase in VAF ≥ 50% was a risk factor for transformation-free survival in the univariate analysis (HR, 11.11; 95% CI, 1.27–97.40; *p* = 0.030), although it lost significance in the multivariate analysis (HR, 17.92; 95% CI, 0.90–358.17; *p* = 0.059). By contrast, a gradual increase in VAF remained an independent predictor of transformation-free survival (HR, 25.23; 95% CI, 1.31–484.77; *p* = 0.032) (Fig. 7). Similarly, an increase in VAF ≥ 50% and a pattern of gradual increase were risk factors for overall survival in the univariate analysis; however, both lost significance in the multivariate analysis (data not shown).

## Discussion

In this study, the mutated *JAK2* allele burden was correlated with neutrophil counts, platelet counts, and LDH levels, supporting that *JAK2*V617F is linked to inflammation and the overall disease burden. Growing evidence suggests that inflammation contributes to the pathophysiology of Ph^−^ MPNs, and that many signs and symptoms in these patients stem from inflammatory activity [18–20]. Here, patients with a higher *JAK2*V617F VAF at diagnosis tended to have a poorer thrombosis-free survival than those with a lower VAF, consistent with earlier reports [10, 11].

Hydroxyurea reduces the *JAK2*V617F allele burden in ET and PV for several years following treatment initiation [21, 22]; however, it typically does not produce a sustained reduction [23, 24]. Similar results have been reported in randomized trials comparing interferon alpha-2b with conventional therapy in patients with PV [25]. Our findings were consistent: the mutated allele burden declined during the first year of hydroxyurea therapy but gradually rebounded thereafter. These observations suggest that hydroxyurea does not lead to major long-term changes in *JAK2*V617F allele burden.

We examined the clinical implications of *JAK2*V617F allele burden changes from two perspectives: individual episodes of increase or decrease and the overall pattern of change over time. Even a single episode of increasing allele burden adversely affected transformation-free and overall survival regardless of the magnitude of the increase. Regarding longitudinal patterns, the transformation-free survival was significantly worse in patients with a gradual increase than in those with a gradual decrease. These results imply that episodic and gradual increases in allele burden confer a higher risk of disease transformation in ET and that an episodic rise often reflects an underlying gradual upward trend. However, a transformation-free survival analysis based on the VAF level at diagnosis could not be performed because few patients with baseline VAF data underwent disease transformation.

In this study, 12 of 87 patients (13.8%) showed a gradual decrease in allele burden and experienced a favorable course, particularly with respect to transformation-free survival; however, this finding has not been previously reported. In a previous study that motivated our interest in serial VAF measurements, the authors observed a progressive increase in 4.4% of 184 patients with ET, with the remaining patients showing a stable allele burden [13]. Differences in the definition of allele burden patterns and duration of follow-up may partly explain the discrepancy in the frequency of gradual (or progressive) increases. Notably, that study did not report a gradual decrease in allele burden in any patient with ET, although a progressive decrease (“unexplained decrease”) was noted in 6% of patients with PV [13]. However, the reasons for these differences remain unclear and require further investigation. In our cohort, *JAK2*V617F was undetectable in two patients treated with hydroxyurea during follow-up, although one patient later experienced a low-level recurrence. A young male patient demonstrated a steady decrease in VAF over 14 years without cytoreductive therapy and ultimately achieved normalization of platelet count. A few case reports have described spontaneous regression of ET [26–28], including one patient with complete molecular remission [28]. Collectively, these observations imply that a subset of patients with ET may achieve molecular remission independent of cytoreductive treatment, warranting further investigation.

This study had some limitations. First, the number of patients included was relatively small. Second, only a subset of patients with *JAK2*V617F-mutated ET diagnosed during the study period was analyzed; not all patients underwent VAF assessment at diagnosis or regular follow-up, which may have introduced selection bias. Third, we did not investigate additional genetic alterations beyond *JAK2*V617F, which could influence clinical outcomes.

Despite these limitations, our results demonstrate that the *JAK2*V617F allele burden changes over time in patients with ET and that these changes are associated with clinical outcomes. Therefore, monitoring of the allele burden is warranted, although the optimal testing interval remains to be determined.

## Data Availability

No datasets were generated or analysed during the current study.

## References

[1] Song IC, Yeon SH, Lee MW, et al. Thrombotic and hemorrhagic events in 2016 World Health Organization-defined Philadelphia-negative myeloproliferative neoplasm. Korean J Intern Med. 2021;36:1190–203. 34289585 10.3904/kjim.2020.634 PMC8435504

[2] Song IC, Yeon SH, Lee MW, et al. Myelofibrotic and leukemic transformation in 2016 WHO-defined Philadelphia-negative myeloproliferative neoplasm. Blood Res. 2022;57:59–68. 35256550 10.5045/br.2021.2021209 PMC8958372

[3] Awada H, Voso MT, Guglielmelli P, Gurnari C. Essential thrombocythemia and acquired von Willebrand syndrome: the shadowlands between thrombosis and bleeding. Cancers (Basel). 2020;12:1746. 10.3390/cancers12071746 PMC7407619 32629973

[4] Stein BL, Martin K. From Budd-Chiari syndrome to acquired von Willebrand syndrome: thrombosis and bleeding complications in the myeloproliferative neoplasms. Blood. 2019;134:1902–11. 31778549 10.1182/blood.2019001318

[5] Rungjirajittranon T, Owattanapanich W, Ungprasert P, Siritanaratkul N, Ruchutrakool T. A systematic review and meta-analysis of the prevalence of thrombosis and bleeding at diagnosis of Philadelphia-negative myeloproliferative neoplasms. BMC Cancer. 2019;19:184. 30819138 10.1186/s12885-019-5387-9 PMC6393965

[6] Reeves BN, Moliterno AR. Thrombosis in myeloproliferative neoplasms: update in pathophysiology. Curr Opin Hematol. 2021;28:285–91. 34183535 10.1097/MOH.0000000000000664

[7] Hsiao HH, Yang MY, Liu YC, et al. The association of JAK2V617F mutation and leukocytosis with thrombotic events in essential thrombocythemia. Exp Hematol. 2007;35:1704–7. 17920754 10.1016/j.exphem.2007.08.011

[8] Carobbio A, Thiele J, Passamonti F, et al. Risk factors for arterial and venous thrombosis in WHO-defined essential thrombocythemia: an international study of 891 patients. Blood. 2011;117:5857–9. 21490340 10.1182/blood-2011-02-339002

[9] Haider M, Gangat N, Lasho T, et al. Validation of the revised International Prognostic Score of Thrombosis for Essential Thrombocythemia (IPSET-thrombosis) in 585 Mayo Clinic patients. Am J Hematol. 2016;91:390–4. 26799697 10.1002/ajh.24293

[10] Bertozzi I, Bogoni G, Biagetti G, et al. Thromboses and hemorrhages are common in MPN patients with high JAK2V617F allele burden. Ann Hematol. 2017;96:1297–302. 28585070 10.1007/s00277-017-3040-8

[11] Guglielmelli P, Loscocco GG, Mannarelli C, et al. JAK2V617F variant allele frequency > 50% identifies patients with polycythemia vera at high risk for venous thrombosis. Blood Cancer J. 2021;11:199. 34897288 10.1038/s41408-021-00581-6 PMC8665926

[12] Koren-Michowitz M, Landman J, Cohen Y, et al. JAK2V617F allele burden is associated with transformation to myelofibrosis. Leuk Lymphoma. 2012;53:2210–3. 22524513 10.3109/10428194.2012.682308

[13] Alvarez-Larran A, Bellosillo B, Pereira A, et al. JAK2V617F monitoring in polycythemia vera and essential thrombocythemia: clinical usefulness for predicting myelofibrotic transformation and thrombotic events. Am J Hematol. 2014;89:517–23. 24458835 10.1002/ajh.23676

[14] Guglielmelli P, Mora B, Gesullo F, et al. Clinical impact of mutated JAK2 allele burden reduction in polycythemia vera and essential thrombocythemia. Am J Hematol. 2024;99:1550–9. 38841874 10.1002/ajh.27400

[15] Arber DA, Orazi A, Hasserjian R, et al. The 2016 revision to the World Health Organization classification of myeloid neoplasms and acute leukemia. Blood. 2016;127:2391–405. 27069254 10.1182/blood-2016-03-643544

[16] Passamonti F, Thiele J, Girodon F, et al. A prognostic model to predict survival in 867 World Health Organization-defined essential thrombocythemia at diagnosis: a study by the International Working Group on Myelofibrosis Research and Treatment. Blood. 2012;120:1197–201. 22740446 10.1182/blood-2012-01-403279

[17] Lee MW, Yeon SH, Ryu H, et al. Volumetric splenomegaly in patients with essential thrombocythemia and prefibrotic/early primary myelofibrosis. Int J Hematol. 2021;114:35–43. 33704663 10.1007/s12185-021-03121-x

[18] Chatain N, Koschmieder S, Jost E. Role of inflammatory factors during disease pathogenesis and stem cell transplantation in myeloproliferative neoplasms. Cancers (Basel). 2020;12:2250. 10.3390/cancers12082250 PMC7463735 32806517

[19] Mendez Luque LF, Blackmon AL, Ramanathan G, Fleischman AG. Key Role of inflammation in myeloproliferative neoplasms: instigator of disease initiation, progression. and symptoms. Curr Hematol Malig Rep. 2019;14:145–53. 10.1007/s11899-019-00508-w PMC7746200 31119475

[20] Lussana F, Rambaldi A. Inflammation and myeloproliferative neoplasms. J Autoimmun. 2017;85:58–63. 28669446 10.1016/j.jaut.2017.06.010

[21] Girodon F, Schaeffer C, Cleyrat C, et al. Frequent reduction or absence of detection of the JAK2-mutated clone in JAK2V617F-positive patients within the first years of hydroxyurea therapy. Haematologica. 2008;93:1723–7. 18728027 10.3324/haematol.13081

[22] Ricksten A, Palmqvist L, Johansson P, Andreasson B. Rapid decline of JAK2V617F levels during hydroxyurea treatment in patients with polycythemia vera and essential thrombocythemia. Haematologica. 2008;93:1260–1. 18519514 10.3324/haematol.12801

[23] Zalcberg IR, Ayres-Silva J, de Azevedo AM, Solza C, Daumas A, Bonamino M. Hydroxyurea dose impacts hematologic parameters in polycythemia vera and essential thrombocythemia but does not appreciably affect JAK2-V617F allele burden. Haematologica. 2011;96:e18-20. 21357711 10.3324/haematol.2010.037846 PMC3046289

[24] Dam MJB, Pedersen RK, Knudsen TA, et al. Data-driven analysis of the kinetics of the JAK2V617F allele burden and blood cell counts during hydroxyurea treatment of patients with polycythemia vera, essential thrombocythemia, and primary myelofibrosis. Eur J Haematol. 2021;107:624–33. 34411333 10.1111/ejh.13700

[25] Gisslinger H, Klade C, Georgiev P, et al. Ropeginterferon alfa-2b versus standard therapy for polycythaemia vera (PROUD-PV and CONTINUATION-PV): a randomised, non-inferiority, phase 3 trial and its extension study. Lancet Haematol. 2020;7:e196-208. 32014125 10.1016/S2352-3026(19)30236-4

[26] Beard ME, Baker BW. Spontaneous remission of essential thrombocythaemia during two consecutive pregnancies. Eur J Haematol. 1989;43:348–9. 2583261 10.1111/j.1600-0609.1989.tb00311.x

[27] Samuelsson J, Swolin B. Spontaneous remission during two pregnancies in a patient with essential thrombocythaemia. Leuk Lymphoma. 1997;25:597–600. 9250833 10.3109/10428199709039050

[28] Otsuka M, Hanada S, Arita K, Ohashi H. Spontaneous regression of essential thrombocythemia with MPL mutation on menopause. Int J Hematol. 2014;99:668–70. 24609764 10.1007/s12185-014-1544-8

